# Tumor-Targeted Fluorescence Imaging and Mechanisms of Tumor Cell-Derived Carbon Nanodots

**DOI:** 10.3390/pharmaceutics14010193

**Published:** 2022-01-14

**Authors:** Taotao Huo, Wenshuai Li, Dong Liang, Rongqin Huang

**Affiliations:** Department of Pharmaceutics, School of Pharmacy, Key Laboratory of Smart Drug Delivery, Ministry of Education, Fudan University, Shanghai 201203, China; 18111030033@fudan.edu.cn (T.H.); 20111030048@fudan.edu.cn (W.L.); dliang@fudan.edu.cn (D.L.)

**Keywords:** carbon nanodots, tumor-targeted fluorescence imaging, LAT1

## Abstract

An ideal cancer diagnostic probe should possess precise tumor-targeted accumulation with negligible sojourn in normal tissues. Herein, tumor cell-derived carbon nanodots (C-CND_U87_ and C-CND_HepG2_) about 3~7 nm were prepared by a solvothermal method with stable fluorescence and negligible cytotoxicity. More interestingly, due to the differences in gene expression of cancers, C-CND structurally mimicked the corresponding precursors during carbonization in which carbon nanodots were functionalized with α-amino and carboxyl groups with different densities on their edges. With inherent homology and homing effect, C-CND were highly enriched in precursor tumor tissues. Mechanistic studies showed that under the mediation of the original configuration of α-amino and carboxyl groups, C-CND specifically bound to the large neutral amino acid transporter 1 (LAT1, overexpressed in cancer cells), achieving specific tumor fluorescence imaging. This work provided a new vision about tumor cell architecture-mimicked carbon nanodots for tumor-targeted fluorescence imaging.

## 1. Introduction

Carbon nanodots (CND) have shown great potential in cancer diagnosis and treatment based on readily available materials, facile synthesis techniques, highly tunable fluorescence properties, and natural biocompatibility [1,2]. With versatile surface functionalization, the promising active targeting CND have been engineered by ligand modification binding to overexpressed receptors in tumor, enhancing the targeted accumulation of CND in tumorous tissues, and decreasing the toxicity in surrounding normal tissues [3,4]. However, functionalization methods inevitably partially destroy the material structure of CND, which might cause the loss of certain intrinsic properties such as compromised fluorescence intensity. Moreover, conjugates reacting with CND via electrostatic forces or π–π interactions, usually exhibit lower stability when facing a tricky internal environment, in particular [1,5,6]. Although introducing some functional groups during synthesis could improve the targeting ability of CND to some extent, more accurate tumor-targeted delivery is still achieved by the post modification strategy in which the complicated synthesis process and incremental particle size could impose limitations on the applications of CND. Thus, carbon nanodots, prepared by the one-step method, with specific and precise tumor-targeting properties, are urgently needed.

For cancer-targeted imaging and drug delivery, differentially upregulated carrier transporters such as large neutral amino acid transporter 1 (LAT1) and glucose transporters on the surface of tumorous cells could be prudently taken into account [7,8]. Among them, LAT1, a sodium and pH-independent transmembrane transporter, is overexpressed in various human cancers to deliver large and neutral amino acids for tumor growth and survival. When knocking down LAT1 with RNA interference, tumor cells exhibit significantly reduced amino acid uptake and poor proliferation [9,10,11]. Meanwhile, LAT1 is overexpressed in the blood–brain barrier (BBB) in which it regulates the transport of amino acids, medicines, and thyroid hormones into brain [12,13]. Several prescription drugs including L-DOPA, baclofen, gabapentin, and melphalan, exhibit strong structural similarity to endogenous substrates of LAT1 in which the unsubstituted α-amino and carboxyl groups modify the side chain of drugs, achieving effective LAT1 binding, and improved the therapeutic effect [11,14]. Recently, carbon nanodots (LAAM CQDs), synthesized by 1,4,5,8-tetraminoanthraquinone and urinary citrate, with paired α-amino and carboxyl groups on their edges, have shown selective and efficient tumor accumulation for imaging with free limitation of origin and location of tumor tissues [13]. This suggests that the design philosophy of structural analogues of LAT1 substrates are not only limited to the prodrug discovery.

Compared with the synthesis of carbon nanodots using non-renewables, the bio-based raw materials regarded as precursors are possibly more popular and sustainable. Both plant- and animal-based carbon sources have been widely explored for CND preparation [15,16,17], and cells, the basic structural and functional unit of organism, are particularly adept at carrying out defined functions within complex environment. With the influence of genes and survival environment, each kind of cell has its own unique protein composition that is critical for their physiological effects. Through protein channels and receptors, cells can contact a wide range of proteins and extracellular matrices, and elegantly manage to execute the specific tasks such as immunologic defense, proliferation, differentiation, and nutritional intake. Moreover, by synthesizing their own proteins, cells maintain intrinsic identities such as adaption to the surroundings, tissue differentiation with various physiological characteristics, cancerous properties, and distinguishing features between species [18,19,20]. When facing the high temperature carbonization, cellular contents such as proteins, biological enzymes, and transporters, could be carbonized prior to dissociation. The original biological effects, such as membrane protein recognition and enzymic catalytic reaction, will not be retained such as cell membrane-coated nanoparticles and tumor exosome delivery systems [21,22]. In contrast, the elemental composition and configuration of carbon nanodots are likely to inherit the elemental structure of biological precursors like carboxyl and amino groups modified LAAM CQDs [13]. Moreover, it is believed that when confronted with cancer, CND prepared by tumor cells, where the composition of surface functional groups and carbon nanodot skeleton are regulated by precursor genes, might exhibit more specific and desirable tumor-targeted capacity than those prepared by the artificial mass ratio regulation of exogenous raw materials.

**Scheme 1:**
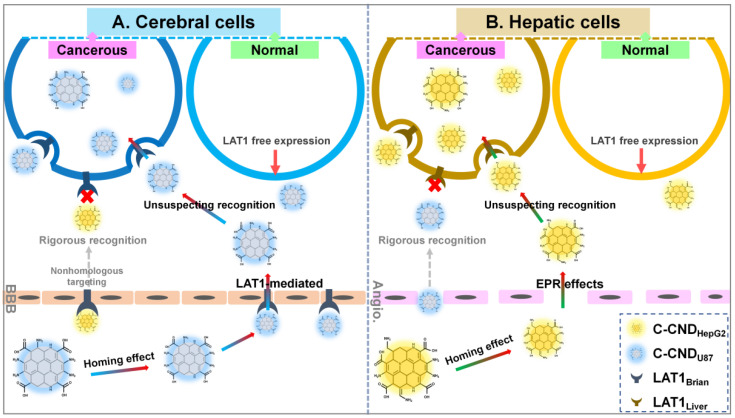
Schematic diagram of in vivo imaging of the tumor cell-derived C-CND. (**A**) C-CND_U87_ mediated fluorescence targeted imaging for glioma across the BBB; (**B**) C-CND_HepG2_ mediated fluorescence targeted imaging for hepatic carcinoma. BBB: Blood–brain barrier, Angio.: Tumor angiogenesis.

Herein, tumor cell-derived carbon nanodots from glioma U87 cells (C-CND_U87_) and hepatoma HepG2 cells (C-CND_HepG2_) were explored for tumor-targeted fluorescence imaging (Scheme 1). Through a one-step direct solvothermal reaction, C-CND prepared by different kinds of tumor cells as precursors showed uniform particle size and stable fluorescence emission. Moreover, with some features retained such as the similar density and configuration of α-amino and carboxyl groups derived from precursors, C-CND owned the just-as-good homing effect and targeted identification of tumor cells. Furthermore, under the mediation of α-amino and carboxyl groups, C-CND specifically bound to LAT1, quickly and preferentially entered their precursor cells, achieving the tumor-targeted accumulation and promising fluorescence imaging.

## 2. Materials and Methods

### 2.1. Cell lines and Animals

Human glioma cell lines (U87 cells), human brain capillary endothelial cell lines (1800 cells), Human hepatocellular carcinoma cell lines (HepG2 cells), and Human hepatocytes of normal cell lines (HL7702 cells) were bought from the Chinese Academy of Science cells Bank (Shanghai, China). U87, 1800, and HepG2 cells were maintained in DMEM-based complete media with FBS (10%), L-glutamine (1%), penicillin (1%), and streptomycin (1%) and cultured in an incubator with 5% CO_2_ at 37 °C. HL7702 cells were provided using RPMI-1640 based complete media with FBS (15%), L-glutamine (1%), penicillin (1%), and streptomycin (1%), and the incubation conditions were the same as those of HepG2. Nude male mice, 7 weeks old, 20~22 g, were bought from the Department of Experimental Animals, Fudan University. All animal experiments were performed as prescribed by the guidelines evaluated and approved by the ethics committee of Fudan University (approval number: 2019–03-YJ-HRQ-01).

### 2.2. Preparation of the Cell-Derived Carbon Nanodots (C-CND)

For preparation of C-CND, the well-grown adherent cells (1.2 × 10^8^) were collected by trypsin-mediated digestion and resuspended by 10 mL anhydrous alcohol in centrifuge tube. After ultrasonic water bath for 1 min, the cell suspension was put into a Teflon-lined autoclave (specification: 20 mL), and after being heated at 200 °C for about 9 h, the result solution was collected and dialyzed with dialysis membranes (44 mm, MWCO = 1000 Da) against pure water for three days to remove the residual solvent. The obtained C-CND were dispersed in water for further usage.

### 2.3. Characterizations

Morphologic characteristics of C-CND were observed using a JEM-2100F Transmission electron microscopy (TEM) with 200 kV acceleration voltage (Tokyo, Japan). For TEM observation, C-CND was dispersed in anhydrous alcohol (10 µg/mL) and supported on an ultrathin carbon-coated copper grid. Surface charge and grain size were measured using Zeta Potential/Particle Sizer Malvern 3600 (Malvern, UK) in which C-CND was diluted with ultra-pure water (10 µg/mL) as the sample. The molecular structure and chemical component contents of C-CND were evaluated by X-ray photoelectron spectroscopy (XPS), Fourier-transform infrared (FT-IR) spectroscopy and ^13^C and ^1^H nuclear magnetic resonance (NMR) spectrum. FT-IR spectroscopy was obtained from the Thermo Nicolet AVATAR 360 FT-IR using the KBr pellet method (Waltham, MA, USA). XPS was conducted by an RBD upgraded PHI-5000C ESCA system with Al Kα radiation (hv = 1486.6 eV) as the X-ray source for excitation (Waltham, MA, USA). ^13^C and ^1^H NMR were performed on a DMX 500 (Bruker) with deuterated chloroform as the solvent. Fluorescence excitation/emission spectra were operated on the Edinburgh FS5 Fluorescence Spectrophotometer (Edinburgh, UK). Ultraviolet spectrum was obtained on a UV-2401PC absorption spectrometer (Melbourne, Australia). Fluorescence-mediated images were obtained using a LSM710 laser confocal scanning microscope (CLSM, Baden-Württemberg, Germany). In vivo and ex vivo fluorescence images were acquired by the IVIS spectrum imaging system of PerkinElmer (Waltham, MA, USA).

### 2.4. Fluorescence Stability

The influences of pH, NaCl, serum (10% fetal bovine serum, FBS, FBS: pure water = 1:10, *v*/*v*), and UV light (ZW14S15W) on the fluorescence stability of C-CND were performed at room temperature. The fluorescence stability in complete media at 37 °C in 5% CO_2_ was also studied. The C-CND (10 µg/mL) were dispersed in pure water with NaCl (2 M Na^+^) or 10% FBS, phosphate buffer solution (PBS) with different pH, or exposed to UV light for 72 h. After that, the emission spectra of different C-CND solutions were detected by Edinburgh FS5 Fluorescence Spectrophotometer with 340 nm excitation. Then, the fluorescence (FL) intensity value at 425 nm (*Em*) was collected and the FL intensity was calculated by the following Equation (1).

FL intensity (%) = FL intensity _72 h_/FL intensity _0 h_ × 100% (1)

### 2.5. BCA Test

In this experiment, 1 × 10^6^ cells were collected and lysed for preparation of the protein sample. After that, 40 µL protein sample (20 µg/mL) and 200 µL BCA working solution were added into 96-well plates. After incubation for 0.5 h at 37 °C, the absorbance of samples at 562 nm was quantified by ELISA.

### 2.6. SDS-PAGE Analysis

In this experiment, 1 × 10^6^ cells were collected and lysed for preparation of the protein sample. Then, the protein sample (0.1 mg/mL), reducing agents (3 µL), and LDS (7.5 µL) were mixed with cell lysis buffer (15 µL total volume). After incubation for 10 min at 70 °C, 10 µL samples were loaded into a 10% NuPAGE Bis-Tris gels with 10 well in MOPS buffer. The electrophoresis was performed at 200 V for 3 h. After that, the SDS-PAGE gel was stained with EZBlueTM gel staining reagent for 0.5 h at room temperature (25 °C) away from light and then washed with 55% aqueous ethanol (5% acetic acid, *v:v*) before taking photos.

### 2.7. Cellular Uptake

U87, 1800, HepG2, and HL7702 cells were planked with lower cell density (10,000 cells/well) in 96 well plate. After adherent growth at 37 °C for 24 h, the media were removed and cell modified wells were washed with PBS for 3 times. For time-dependent cellular uptake, 80 µg/mL of C-CND diluted in complete media were added into plates. At predetermined time point (0.5, 1, 1.5, and 2 h), the fluorescence intensity of C-CND within cells was observed using LSM710 CLSM. For concentration dependent uptake manner, different C-CND diluted in complete media were added into the plate. After co-incubation for 2 h, cells were washed with PBS, and fixed by paraformaldehyde (4%). The fluorescence intensity of C-CND was observed by CLSM. Meanwhile, cell-specific uptake was carried out by incubation of different cells with different C-CND (80 µg/mL). Then the cells were fixed by paraformaldehyde (4%) before collecting the images. For the study of mechanism of cellular uptake, cells were planked and cultured as above. After adherent growth for 24 h, 10 mM amino acids including Glycine (Gly), phenylalanine (Phe), tryptophan (Trp), and leucine (Leu, the endocytosis inhibitors including PhAsO (30 µM), filipin (500 nM) and colchicine (12 µg/mL) were added and co-incubated with cells for another 0.5 h, respectively. After that, cells were incubated with C-CND (80 µg/mL) containing the same number of amino acids and inhibitors for another 2 h, sequentially. Then, the plate wells were washed using PBS, and cell were fixed by paraformaldehyde (4%). The fluorescence uptake images were collected by a Leica fluorescent microscope (DMI4000B, Hesse-Darmstadt, Germany).

### 2.8. Subcellular Localization of C-CND

Cancer cells (U87 and HepG2) were seeded in confocal dishes at a density of 1 × 10^4^ cells/well, respectively. After incubation for 24 h, the MitoTracker^®^ Red FM (581/644 nm) and LysoTracker^®^ Green DND-26 (504/511 nm) were added where 30 min incubation for mitochondria staining and 1 h incubation for lysosome staining. After that, 80 µg/mL C-CND was added and incubated for another 0.5 and 2 h at 37 °C. Then, cells were rinsed 3 times with PBS and fluorescence images were collected by a LSM710 CLSM.

### 2.9. Sample Preparation

6 × 10^6^ cells were collected and lysed by ultrasonication. After cryogenic centrifugation (12,000 rpm/15 min), 800 µL MeOH containing 40 µg/mL tridecanoic acid, and 200 µL supernatant were added into tubes and mixed thoroughly by using vortex. After cryogenic centrifugation (12,000 rpm/min) for 15 min, 600 µL supernatant was collected and the samples were dried under a nitrogen stream at 40 °C. Then 50 µL methoxyamine pyridine solution (15 mg/mL) was added for oximation reaction and 40 µL MSTFA was added for silylation reaction (containing 1% chlorotrimethylsilane). After completion of the derivatization reaction, the solution was centrifuged and the 50 µL supernatant was collected for gas chromatography-mass spectrometry (GC-MS) analysis.

### 2.10. Construction of Tumor-Bearing Mice Models

For U87 orthotopic glioma models, the nude mice under a 10% chloral hydrate mediated general anesthesia were challenged with U87 cells (5 × 10^5^) on the right caudatoputamen by using a standard mice stereotactic fixation equipment with an adaptor (M5091). When the procedure was complete, the pinhole in the skull was sealed and the skin of the wound was stitched, and after 2 weeks, the animal models were used for further research. For the subcutaneous model of hepatic carcinoma, mice were challenged with HepG2 cells (2 × 10^6^) on their right flank. After 10 days, mice with tumors of about 100 mm^3^ were selected for following studies.

### 2.11. In Vivo Tissue Distribution

Tumor-bearing models of nude mice including U87 orthotopic glioma and HepG2 subcutaneous hepatoma were administered with 100 µL C-CND (50 mg/kg) through the tail vein after an overnight fast. At the estimated time points (0, 1, 2, 3, and 4 h), the trends of fluorescence accumulation in mice were observe using an IVIS Spectrum in vivo imaging system (Caliper, MA, USA) under a general anesthetic (3% isoflurane flow). At the 3 h post-injection, one mouse of each group was sacrificed. Tumor tissues and main organs were collected and imaged for the distribution of C-CND in vivo.

### 2.12. Study on the Relationship between C-CND and Tumor Proliferation

U87 cells and HepG2 cells were planked with 1 × 10^4^ cells/well in 96-well plates, respectively. After cultivation at 37 °C for 24 h, complete media, DMEM with FBS/glucose free media, and 4 mg/mL of C-CND DMEM media were added into well plates, respectively, for another 24 h incubation. Then, the cells were washed 3 times with PBS, 100 µL CCK 8 test media was added to each well and incubated for another 1 h at 37 °C. The absorbance (A450) was measured on a microplate reader. Accordingly, the cell survival rate was calculated.

### 2.13. Tumorigenicity Study

For study of tumorigenicity of C-CND_U87_ and C-CND_HepG2_, mice were challenged with tumor cells (2 × 10^6^) and corresponding precursor-based C-CND (200 mg/kg) on their right flank, respectively. After 15 days of subcutaneous tumor inoculation, the mice were kept under anesthesia and took photos.

### 2.14. Statistical Analysis

All sample data were collected at least in triplicate. The data were analyzed by the Student’s *t*-test and ANOVA, and the statistic difference including *p* < 0.05 (*) and 0.01 (**) were considered as significant.

## 3. Results and Discussion

**Figure 1:**
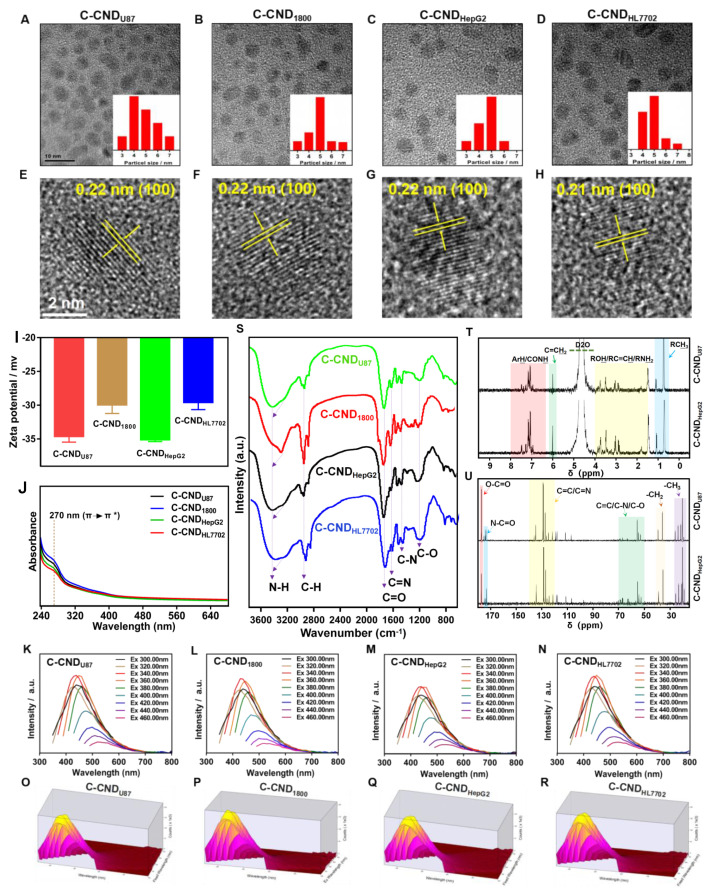
(**A**−**D**) TEM images (Bar = 10 nm), (**E**−**H**) HRTEM images (Bar = 2 nm), (**I**) Zeta potentials, (**J**) UV-vis spectra, (**K**−**R**) Excitation-dependent emission (*Em*) spectra, and (**S**) FTIR patterns of different C-CND. (**T**) ^1^H-NMR and (**U**) ^13^C-NMR spectra of C-CND_U87_ and C-CND_HepG2_. Data are represented as mean ± SD (*n* = 3). Yellow arrows: the d-spacing of C-CND.

Two types of cancerous cells including human glioma cell line (U87) and hepatoma cell line (HepG2) and corresponding normal cell lines including human astrocyte cell line (1800) and hepatocyte cell line (HL7702) were used for C-CND preparation. As shown in Figure 1A−D, all the C-CND held highly uniform sphere-like morphology with particle size around 3~7 nm, which matched with the result of dynamic light scattering (DLS) (Supplementary Materials Figure S1, Supporting Information). HRTEM images of C-CND showed a graphitic carbon core with well-resolved lattice fringes in which C-CND_U87_, C-CND_1800_, and C-CND_HepG2_ showed the typical (100) plane (d-spacing = 0.22 nm), and C-CND_HL7702_ showed the typical (100) plane (d-spacing = 0.21 nm) (Figure 1E−H).

**Figure 2:**
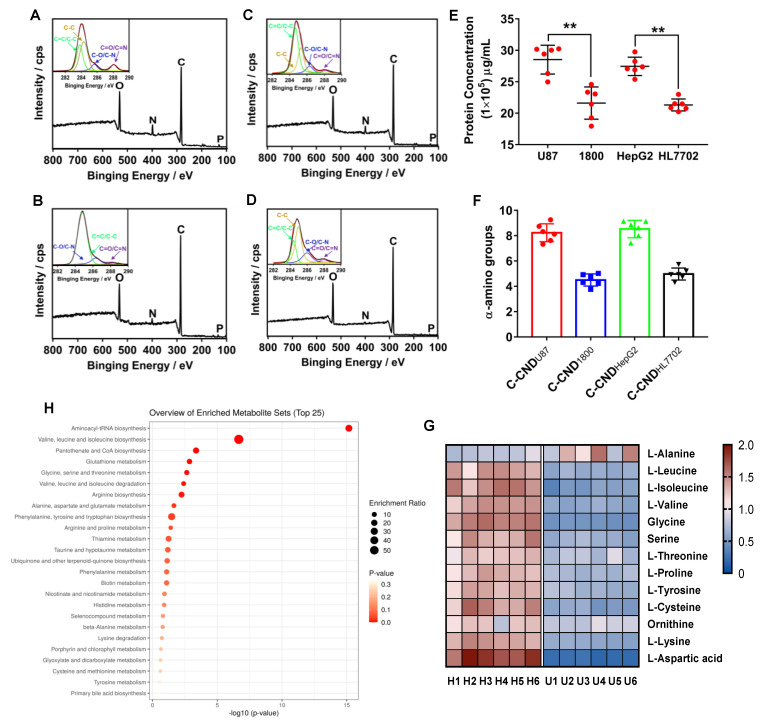
XPS patterns of: (**A**) C-CND_U87_, (**B**) C-CND_1800_, (**C**) C-CND_HepG2_, and (**D**) C-CND_HL7702_. Insert: P1s spectra of corresponding C-CND_._ (**E**) BCA assay of the total protein content of different types of cells. Data are represented as mean ± SD (*n* = 6). (**F**) The number of α-amino groups on the different C-CND. Data are represented as mean ± SD (*n* = 6). (**G**) The amino acid analysis in cancer cell lysates, H: HepG2 cell, U: U87 cell. (**H**) The metabolic pathways associated with amino acids of Figure 2G. Notes: ** *p* < 0.01.

The Zeta potential analysis (Figure 1I) showed the negative charge nature on the surface of C-CND (−34.8 mV for C-CND_U87_, −30.2 mV for C-CND_1800_, −35.2 mV for C-CND_HepG2_, and −29.8 mV for C-CND_HL7702_). Then, the optical fluorescence spectra of C-CND were shown in Figure 1J, all of which exhibited a typical UV absorption of carbon nanodots at 270 nm. Moreover, the stronger fluorescence-emission of the C-CND was observed when excited with ultraviolet irradiation light between 300 and 400 nm (Figure 1K−R). The fluorescence was stable when exposed to media with different pH, ionic strength, 10% FBS, complete media or UV irradiation (Supplementary Materials Figures S2–S7, Supporting Information). The chemical composition of C-CND was studied by X-ray photoelectron spectroscopy (XPS), Fourier-transform infrared (FT-IR) spectroscopy, and ^13^C and ^1^H nuclear magnetic resonance (NMR) spectrum. The characterization absorption peak at 3270 cm^−1^ and 3410 cm^−1^ (N–H), 1710 cm^−1^ (C = O), 1630 cm^−1^ (C = N), and 1350 cm^−1^ (C–N) and 1160 cm^−1^ (C–O) suggested that the free carboxyl and amino groups existed at the edges of C-CND (Figure S1), which was necessary for the recognition and binding of the substrate and the LAT1 receptor [13]. In the ^1^H-NMR spectra (Figure 1T and Supplementary Materials Figures S8–S11, Supporting Information), the signal peaks appearing at 2.50~3.8 ppm corresponded to α-amino protons. Moreover, in the ^13^C-NMR spectra (Figure 1U and Supplementary Materials Figures S12–S15, Supporting Information), the peaks appearing at ~176 ppm, ~173 ppm, and 50~70 ppm were associated with O-C = O, N-C = O, and C-N, respectively, and all the spectra of C-CND possessed four typical peaks of the C, O, N, and P binding energies, indicating that they belonged to the C-CND doped with nitrogen and phosphorus (Figure 2A–D). Among them, the corresponding average elemental content values were 79.11%, 15.04%, 3.82%, and 1.47% for C-CND_U87_ (Figure 2A); 78.87%, 17.85%, 1.93%, and 0.73% for C-CND_1800_ (Figure 2B); 74.96%, 16.26%, 6.75%, and 1.71% for C-CND_HepG2_ (Figure 2C); and 78.48%, 14.46%, 5.21%, and 1.46% for C-CND_HL7702_ (Figure 2D), respectively. The deconvoluted C1s spectra of C-CND revealed that carbon atoms included graphitic or aliphatic carbon (C = C/C-C, 284.4~284.7 eV) and oxygenated or nitrous carbon (C-N/C-O, 286.1~286.4 eV; C = N/C = O, 288.2~288.6 eV) (Figure 2A−D). The O1s spectra of C-CND further verified the presence of oxygenated carbon with typical O-C peak at 531.4~531.8 eV and C = O peak at 532.8~533.3 eV (Supplementary Materials Figure S16, Supporting Information) and the deconvoluted N1s spectra of C-CND showed 3 peaks at 399.1~399.3 eV, 399.6~399.8 eV, and 400.2~401.9 eV, which corresponded to C-N-C, N-C_3_, and N-H, respectively (Supplementary Materials Figure S16, Supporting Information). Although each element contains a proper peak, each peak had a different amount of carbon at different C-CND, and the presence of many complex and different ratios of bonds, which preserved from the precursors during carbonization between C-CND, would affect configuration, composition, and biological applications of carbon nanodots. More interestingly, P2p3/2 peak at 133.2~133.5 eV or P2p1/2 peak at 134.1~134.4 eV, which also was discrepant in different C-CND, in the deconvoluted P2p spectra, indicated that related components of cell membranes were involved during the formation of C-CND (Supplementary Materials Figure S16, Supporting Information) [23], which might regulate the targeted delivery properties of C-CND. All these results thus indicated that 3~7 nm of tumor cell-derived CND with unsubstituted α-amino and carboxyl groups on their edges were successfully prepared. Importantly, the existence of functional groups with different species and densities on the surface might endow C-CND with different targeting characteristics.

To further explore the molecular structure and chemical composition of C-CND, the protein composition and metabolic amino acids in precursors were studied by BCA, SDS-PAGE, and GC-MS, respectively. As shown in Figure 2E, with the same number of cells, the total protein content of tumorous cells was significantly higher than that of corresponding normal cells, indicating that tumors did need more nutrients than normal tissues for survival. Moreover, the result of SDS-PAGE (Supplementary Materials Figure S17, Supporting Information) showed that the content of protein with the same molecular weight was different between cells, such as the proteins about 28 kDa, 63 kDa, and 180 kDa. This self-difference in protein expression between cells might endow C-CND more selectivity during the carbonization. Considering that LAT1 mainly imported the large and neutral amino acids, the metabolites of amino acids in cancer cell lysates that came from the protein components, and also played the role as the precursor of C-CND, were then studied. As shown in Figure 2G, 13 amino acids as the high-affinity substrates of LAT1 were detected in both U87 and HepG2 cells in which 10 including glycine, alanine, valine, leucine, isoleucine, proline, serine, tyrosine, cysteine, threonine were neutral amino acids and three including aspartic acid, ornithine, and lysine were large amino acids [7,9,24,25]. Compared with U87, the level of L-Alanine in HepG2 was the lowest while the level of L-Aspartic acid was the highest. Meanwhile, due to the respective gene-mediated metabolic pathways of cancer cells, the content of the same type of amino acid in lysates of U87 and HepG2 was different (Figure 2H and Supplementary Materials Figure S18, Supporting Information). This difference might result in the C-CND’s unique cellular affinity such as the effortless identification and the guardless endocytosis when C-CND met their precursors. Therefore, under the mediation of the genes (Supplementary Materials Figure S18, Supporting Information), with the same type of nutrients/elements provided, different cells might make appropriate choices to each nutrient/element according to their own growth and proliferation characteristics to meet their own survival needs. C-CND derived from different types of cells were likely to inherit their own unique style in terms of the composition and configuration just like when they were alive, and this unique style might allow C-CND to be recognized and taken up by their precursor cells preferentially when compared to other carbon nanodots. Subsequently, the number of amino groups on the edges of each C-CND was studied by the classical ninhydrin reaction in which the α-amino groups can react with ninhydrin to form the purple-colored product diketohydrindylidene and then produce an absorption at 570 nm [13]. As shown in Figure 2F and Supplementary Materials Figure S19, Supporting Information, treated with ninhydrin, C-CND generated a new adsorption peak at 570 nm, indicating the presence of α-amino groups on the edges of C-CND. A calculation based on the correlation between amino groups and absorption revealed that there were about 8.21% α-amino groups for 1 mg C-CND_U87_, 4.24% α-amino groups for 1 mg C-CND_1800_, 8.76% α-amino groups for 1 mg C-CND_HepG2_, and 4.66% α-amino groups for 1 mg C-CND_HL7702_, respectively (Figure 2F). These results indicated that C-CND with the same morphology might have different properties through different ratios and compositions of elements provided by different precursors, and these surface residues with different α-amino groups, together with tumor homology and homing effects, would make C-CND structurally mimic their corresponding precursor cells. Such C-CND are likely to have unique targeting properties originated from precursors, which might make a significant difference in C-CND-mediated cancer imaging.

**Figure 3:**
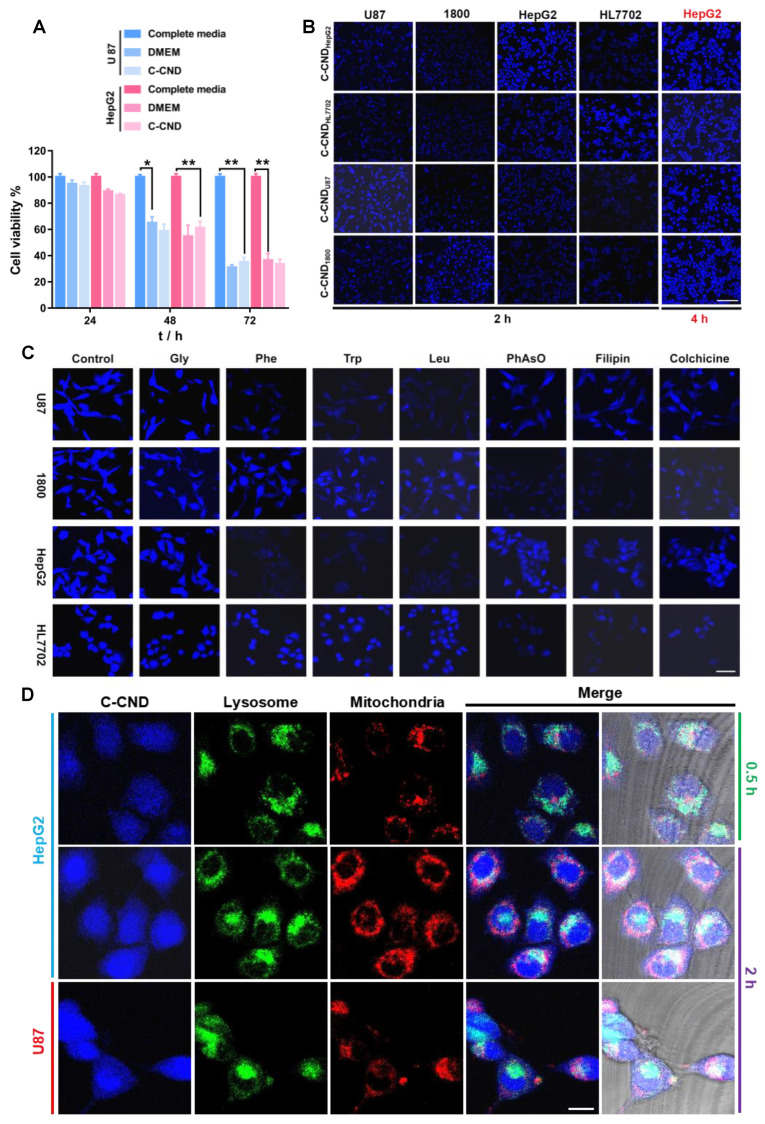
(**A**) Cell viability of U87 cells and HepG2 cells incubated with complete media, DMEM (FBS and glucose free media) and 4 mg/mL of C-CND dispersed in DMEM (U87 cells incubated with C-CND_U87_, HepG2 cells incubated with C-CND_HepG2_) for varied time periods. Data are represented as mean ± SD (n = 4). Confocal fluorescence images of tumorous (HepG2 and U87) and normal (1800 and HL_7702_) cells incubated with different C-CND (80 µg/mL) for (**B**) varied time periods (Bar = 100 µm) and (**C**) being pretreated with Gly, Phe, Trp, Leu, PhAsO, filipin, and colchicine, followed by incubation with corresponding C-CND (Bar = 50 µm). (**D**) The subcellular localization of the C-CND (80 µg/mL) with different organelles. Green: lysosome. Red: mitochondria. Blue: C-CND. Bar = 10 µm. Notes: * *p* < 0.05; ** *p* < 0.01.

The excellent C-CND with special structures and properties could be exploited for biological fluorescence imaging. The CCK-8 test showed that during a single incubation for 24 h, C-CND_U87_ and C-CND_HepG2_ exhibited no obvious cytotoxicity to tumor cells (Figure 3A). Compared with cell viability in DMEM (FBS and glucose free media), C-CND did not provide the nutrition and energy for cell proliferation and growth, indicating that cells after the solvothermal reaction were just CND with targeting properties. Moreover, the tumorigenicity study further showed that neither C-CND_U87_ nor C-CND_HepG2_ had the ability to form tumors (Supplementary Materials Figure S20, Supporting Information), which also suggested the good biosafety of C-CND. The study of cellular uptake showed that C-CND was rapidly taken up by precursor cells in concentration- and time-dependent manner (Figure 3B and Supplementary Materials Figures S21 and S22, Supporting Information) while there was a time delay of uptake in other non-progenitor cells and this variability decreased over time. This indicated that C-CND with different surface characteristic and precursors could show the specific homing effect in which the precursor cells were the first choice (Figure 3B). The mechanism of cellular uptake of C-CND was also studied. As shown in Figure 3C, in contrast to glycine (Gly, non-substrate of LAT1), the uptake of C-CND_U87_ and C-CND_HepG2_ were apparently inhibited by the high-affinity substrates of LAT1 (phenylalanine/Phe, tryptophan/Trp, and leucine/Leu) while the cellular uptake of C-CND_1800_ and C-CND_HL7702_ had no remarkable effect [11,14]. The main causes of this effect were the over expression of LAT1 in tumor cells while negligible expression in normal tissues and the varying densities of α-amino and carboxyl groups on the surface of each C-CND. C-CND (C-CND_U87_ and C-CND_HepG2_) with high-density of amino and carboxyl groups were more likely to be recognized and absorbed by tumor cells [13,14]. The inhibition study using PhAsO (clathrin pathway inhibitor), filipin (caveolae pathway inhibitor), and colchicine (micropinocytosis inhibitor) showed that the cellular uptake of C-CND in normal cells was mainly mediated by the clathrin-mediated endocytosis, caveolae-mediated endocytosis and macropinocytosis [26,27,28,29]. For cancer cells, high expression of LAT1 makes up for the deficiency of other uptake patterns. Thus, when pretreated with PhAsO, filipin, and colchicine, the cellular uptake of C-CND_U87_ and C-CND_HepG2_ was not significantly affected. Theoretically, LAT1 is a sodium independent transmembrane transporter without ATP consumption while clathrin-mediated endocytosis, caveolae-mediated endocytosis, and macropinocytosis belong to the ATP dependent transmembrane transporters [14,30], and tumors that never get enough nutrients and energy would not hesitate to transport nutrients through LAT1. Thus, it is speculated that both the density difference in α-amino and carboxyl groups on the surface of C-CND and upregulated expression of LAT1 in cancer cells lead to selective uptake of C-CND in tumor cells. Furthermore, the study of the intracellular behavior of C-CND_U87_ and C-CND_U87_ showed that C-CND could escape from the lysosomes and mitochondria (Figure 3D).

**Figure 4:**
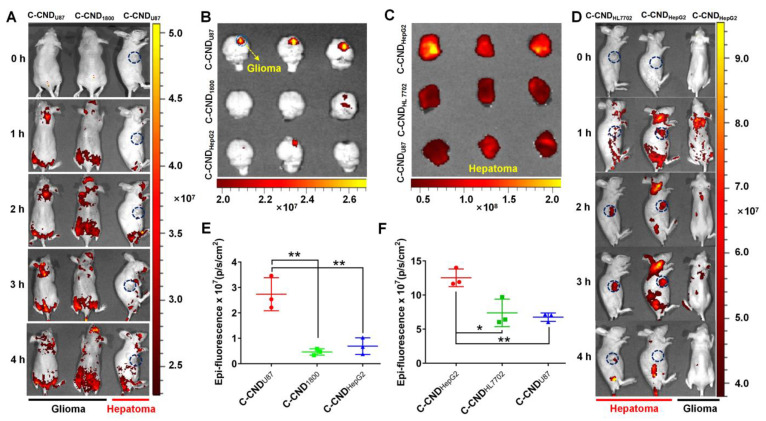
(**A**) Living imaging of orthotopic U87 glioma-bearing mice after intravenous injection of C-CND_U87_ and C-CND_1800_, and the subcutaneous hepatoma-bearing mice after intravenous injection of C-CND_U87_ at different time periods. (**B**) Ex vivo imaging of brains of U87 mediated orthotopic glioma-bearing mice collected at 3 h post intravenous injection of C-CND_U87_, C-CND_1800_, and C-CND_HepG2_ (*n* = 3). (**C**) Ex vivo imaging of tumors of hepatoma-bearing mice after 3 h intravenous injection of C-CND_HepG2_, C-CND_HL7702_, and C-CND_U87_ (*n* = 3). (**D**) Living imaging of the subcutaneous hepatoma-bearing mice after intravenous injection of C-CND_HepG2_ and C-CND_HL7702_, and U87 glioma-bearing mice after intravenous injection of C-CND_HepG2_ at several times. Quantified fluorescence intensity of gliomas of orthotopic U87 glioma-bearing mice collected after 3 h intravenous injection of C-CND_U87_, C-CND_1800_, and C-CND_HepG2_ (**E**) and tumors of hepatoma-bearing mice after 3 h intravenous injection of C-CND_HepG2_, C-CND_HL7702_, and C-CND_U87_ (**F**) (*n* = 3). Notes: * *p* < 0.05; ** *p* < 0.01.

Based on the favorable fluorescence properties and specific tumor targeting ability, C-CND were used for in vivo tumor imaging in the orthotopic glioma-bearing mice and the subcutaneous hepatoma-bearing mice. As shown in Figure 4A,D, the highest fluorescence imaging was observed at 3 h post-injection of C-CND, which showed an extended retention time in tumor tissues compared to the carbon nanodots that previously prepared from the artificial proportioned acellular materials [31,32]. Meanwhile, the accumulation of C-CND to precursor tumors was much higher and longer than that of non-precursor tumors and normal tissues (Figure 4B,C, and Supplementary Materials Figure S23, Supporting Information). Due to the specific expression of LAT1 in the BBB and glioma [7], C-CND_U87_ with higher density of α-amino and carboxyl groups was capable of penetrating across the BBB and achieve more brain tumor accumulation, which thus showed a much stronger fluorescence intensity at glioma than that of C-CND_1800_ (Figure 4B,E). Moreover, with gene-mediated inherent homology and homing effect, C-CND_U87_ preferentially accumulated in glioma with substantially higher signal than that of C-CND_HepG2_. Although the functional group densities of the tumor-originated carbon nanodots were similar, the differentiated gene expression between U87 and HepG2 cells made the types and contents of proteins of the corresponding C-CND different (Supplementary Materials Figures S17 and S18, Supporting Information). Thus, the α-amino and carboxyl groups of the same density were provided by different protein substrates. These C-CND no longer had the biological effects such as carcinogenicity or the provision of nutrients for cells. However, the original residues and the basic configuration were partly maintained after carbonization, which allowed cancer-originated carbon nanodots to be easily recognized and ingested by precursor tumors under the mediation of LAT1 than those CND prepared by the artificial materials. With the same theory, when intravenously injected into hepatoma-bearing mice, due to the difference in α-amino and carboxyl groups, C-CND_HepG2_ showed a brighter fluorescence signal within hepatoma than that of C-CND_HL7702_, and homologous C-CND_HepG2_ was more popular with hepatic tumor tissue than that of C-CND_U87_ (Figure 4C,D,F). Taken together, these results indicate that the tumor cell-derived C-CND could be a new candidate for cancer diagnosis as well as for imaging-mediated oncotherapy with high specificity and efficiency.

## 4. Conclusions

Inspired by tumor cell-derived nanosystems and LAT1-mediated prodrugs, the tumor cell-derived CND (C-CND_U87_ and C-CND_HepG2_) were prepared by a facile solvothermal method with 3~7 nm particle size, possessing stable fluorescence with low cytotoxicity. More interestingly, due to the differences in gene expression of cancers, C-CND structurally mimicked the corresponding precursor after carbonization and functionalized with different densities of α-amino and carboxyl groups on their edges. With the inherent homology and homing effect, C-CND could enrich in tumor tissues, and under the mediation of LAT1, C-CND completed the targeted accumulation within tumor, achieving a specific precursor tumor fluorescence imaging. This work provided a new vision about the carbon nanodots for tumor-targeted fluorescence imaging.

## Supplementary Materials

The following are available online at https://www.mdpi.com/article/10.3390/pharmaceutics14010193/s1, Figure S1: Particle size distribution curves of C-CND determined by DLS; Figure S2: The normalized FL intensity of C-CND_U87_ (a) and C-CND_1800_ (b) upon the exposure to PBS solutions with different pH for 0 h and 72 h, Figure S3: The normalized FL intensity of C-CND_HpeG2_ (a) and C-CND_HL7702_ (b) upon the exposure to PBS solutions with different pH for 0 h and 72 h, Figure S4: The normalized FL intensity of C-CND upon the exposure to Na^+^ solution (2 M) for 0 h and 72 h, Figure S5: The normalized FL intensity of C-CND upon the exposure to pure water containing 10% FBS (*v*/*v*) for 0 h and 72 h, Figure S6: The normalized FL intensity of C-CND upon the exposure to UV excitation for 0 h and 72 h, Figure S7: The normalized FL intensity of C-CND upon the exposure to complete media at 37 °C in 5% CO_2_ for 0 h and 72 h, Figure S8: ^1^H-NMR spectrum of C-CND_U87_, Figure S9: ^1^H-NMR spectrum of C-CND_1800_, Figure S10: ^1^H-NMR spectrum of C-CND_HepG2_, Figure S11: ^1^H-NMR spectrum of C-CND_HL7702,_ Figure S12: ^13^C-NMR spectrum of C-CND_U87_. Solvent: D_2_O, Figure S13: ^13^C-NMR spectrum of C-CND_1800_. Solvent: D_2_O, Figure S14: ^13^C-NMR spectrum of C-CND_HepG2_. Solvent: D_2_O, Figure S15: ^13^C-NMR spectrum of C-CND_HL7702_. Solvent: D_2_O, Figure S16: O1s, N1s and P1s spectra of C-CND_U87_, C-CND_1800_, C-CND_HepG2_ and C-CND_HL7702_, respectively, Figure S17: The SDS-PAGE analysis of proteins of U87 cell, 1800 cell, HepG2 cell and HL7702 cell, Figure S18: The differentiated mRNA expression in U87 and HepG2 cells, Figure S19: UV spectra of histidine after ninhydrin reaction, Figure S20: Tumorigenicity study of C-CND_U87_ and C-CND_HepG2_, Figure S21: Confocal fluorescence images of U87, 1800, HepG2 and HL7702 cells incubated with varied concentrations of C-CND for 2 h under 405 nm excitation, Figure S22: Confocal fluorescence images of U87, 1800, HepG2 and HL7702 cells incubated with 80 µg/mL C-CND for varied time periods under 405 nm excitation, Figure S23: Ex vivo imaging of main organs of tumor-bearing mice (the subcutaneous hepatoma-bearing mice and orthotopic U87 glioma-bearing mice) collected after 3 h intravenous injection of C-CND_HepG2_ and C-CND_HL7702_.
