# Supplementary material for: Tumor-Targeted Fluorescence Imaging and Mechanisms of Tumor Cell-Derived Carbon Nanodots
Source: Pharmaceutics. 2022 Jan 14;14(1):193. doi: 10.3390/pharmaceutics14010193 (PMC8778872; doi:10.3390/pharmaceutics14010193)
Supplement: Supplementary file 1 [file pharmaceutics-14-00193-s001.zip › pharmaceutics-1496278-supplementary.pdf]

pharmaceutics

Supplementary Materials: Tumor-Targeted Fluorescence Imaging and Mechanisms of Tumor Cell-Derived Carbon Nanodots

Taotao Huo, Wenshuai Li, Dong Liang and Rongqin Huang

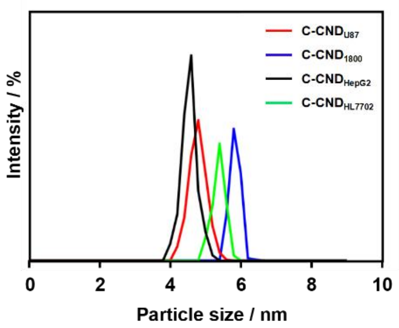

Figure S1. Particle size distribution curves of C-CND$_{U87}$, C-CND$_{1800}$, C-CND$_{HepG2}$ and C-CND$_{HL7702}$ determined by DLS.

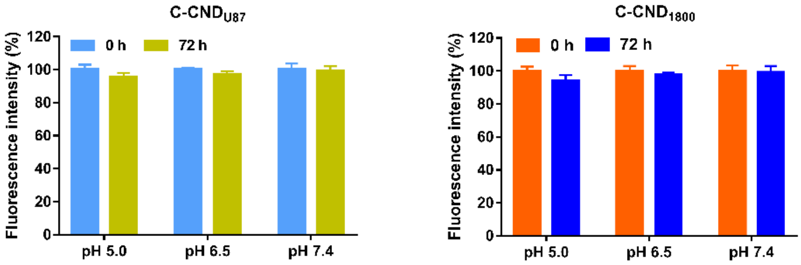

(**a**) (**b**)

Figure S2. The normalized FL intensity of C-CND$_{U87}$ (**a**) and C-CND$_{1800}$ (**b**) upon the exposure to PBS solutions with different pH for 0 h and 72 h. Data are represented as mean ± SD (n = 3).

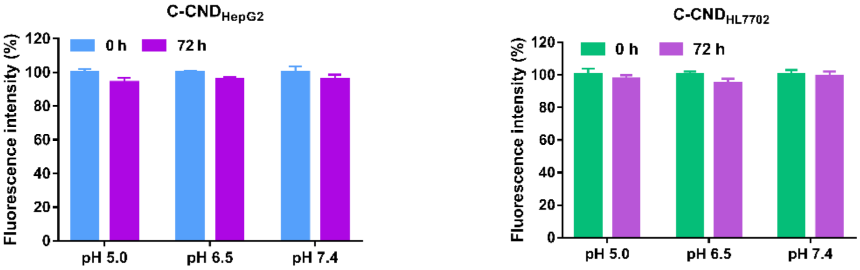

(**a**) (**b**)

Figure S3. The normalized FL intensity of C-CND$_{HpeG2}$ (**a**) and C-CND$_{HL7702}$ (**b**) upon the exposure to PBS solutions with different pH for 0 h and 72 h. Data are represented as mean ± SD (n = 3).

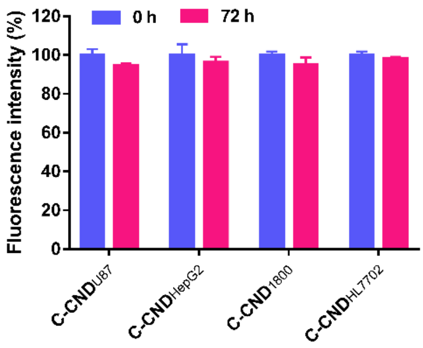

Figure S4. The normalized FL intensity of C-CND upon the exposure to Na^{+} solution (2 M) for 0 h and 72 h. Data are represented as mean ± SD (*n* = 3).

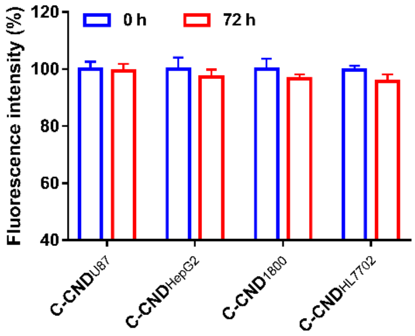

Figure S5. The normalized FL intensity of C-CND upon the exposure to pure water containing 10% FBS (*v*/*v*) for 0 h and 72 h. Data are represented as mean ± SD (*n* = 3).

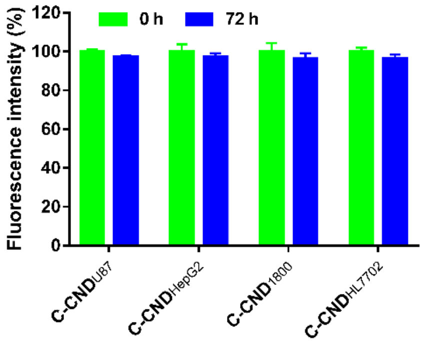

Figure S6. The normalized FL intensity of C-CND upon the exposure to UV excitation for 0 h and 72 h. Data are represented as mean ± SD (*n* = 3).

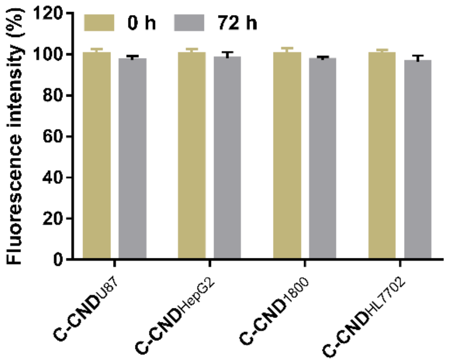

Figure S7. The normalized FL intensity of C-CND upon the exposure to complete media at 37 ℃ in 5% CO_2 for 0 h and 72 h. Data are represented as mean ± SD (*n* = 3).

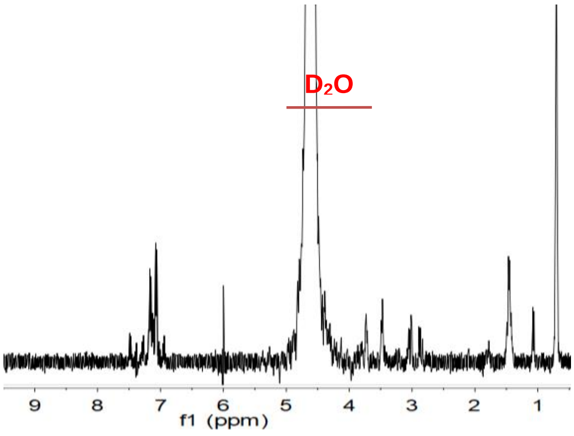

Figure S8. ^{1}H-NMR spectrum of C-CND$_{U87}$.

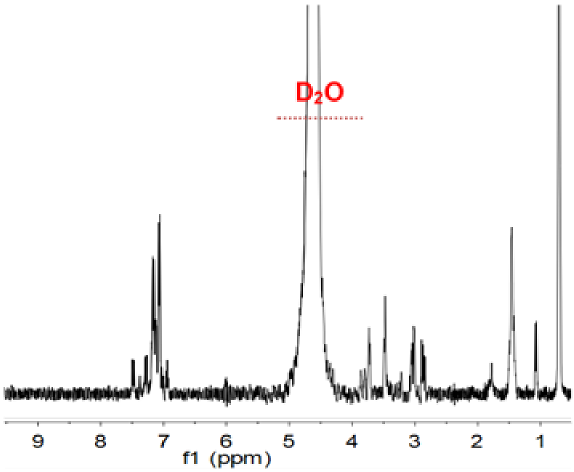

Figure S9. ^{1}H-NMR spectrum of C-CND$_{1800}$.

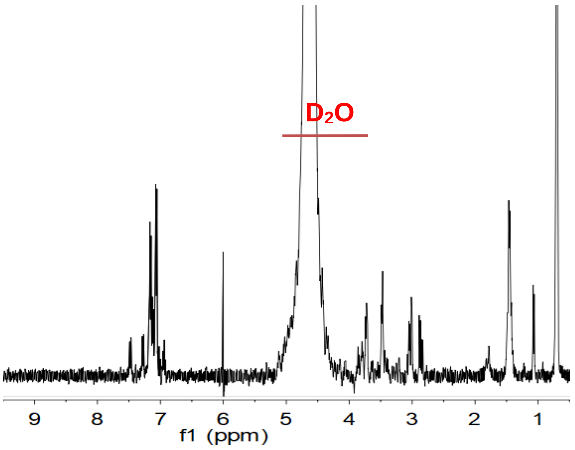

Figure S10. ^{1}H-NMR spectrum of C-CND$_{HepG2}$.

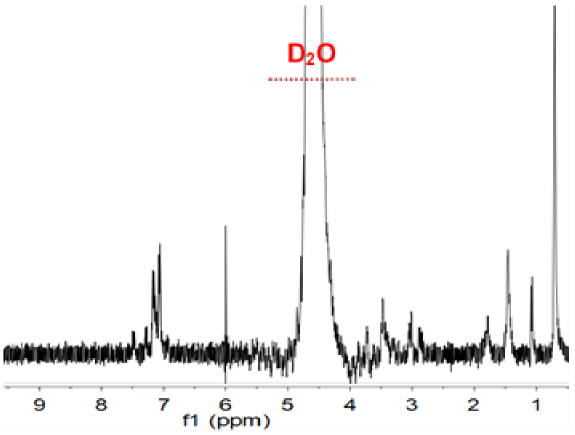

Figure S11. ^{1}H-NMR spectrum of C-CND$_{HL7702}$.

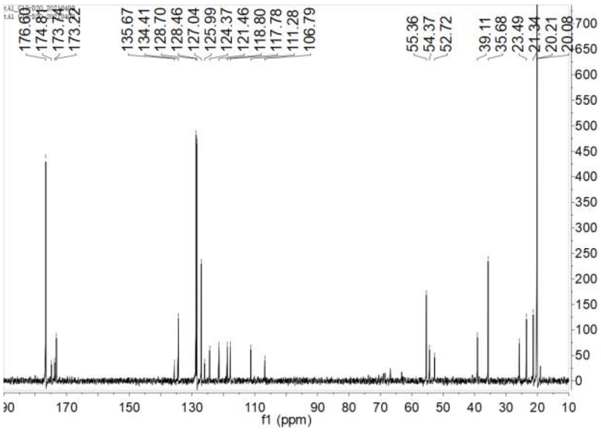

Figure S12. ^{13}C-NMR spectrum of C-CND$_{U87}$. Solvent: D_2O.

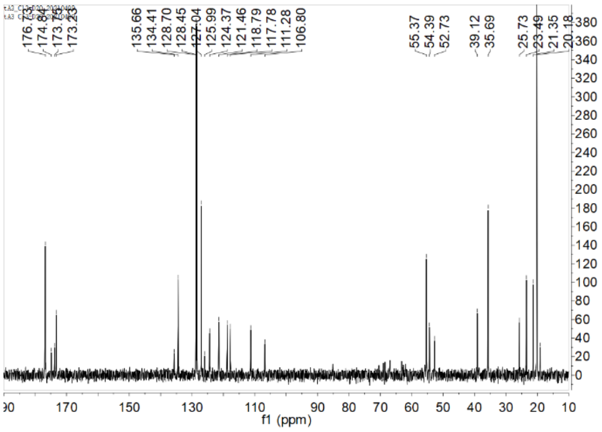

Figure S13. ^{13}C-NMR spectrum of C-CND$_{1800}$. Solvent: D_2O.

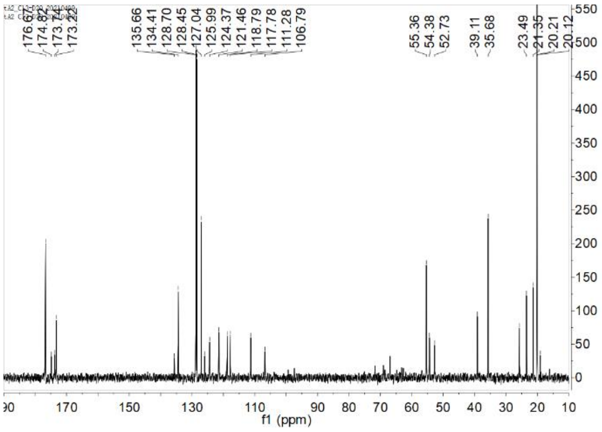

Figure S14. ^{13}C-NMR spectrum of C-CND$_{HepG2}$. Solvent: D_2O.

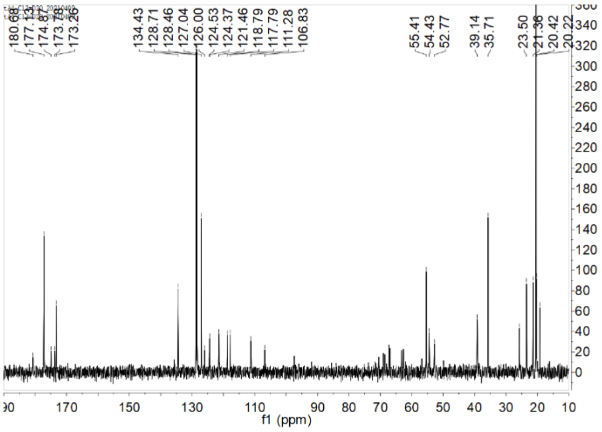

Figure S15. ^{13}C-NMR spectrum of C-CND$_{HL7702}$. Solvent: D_2O.

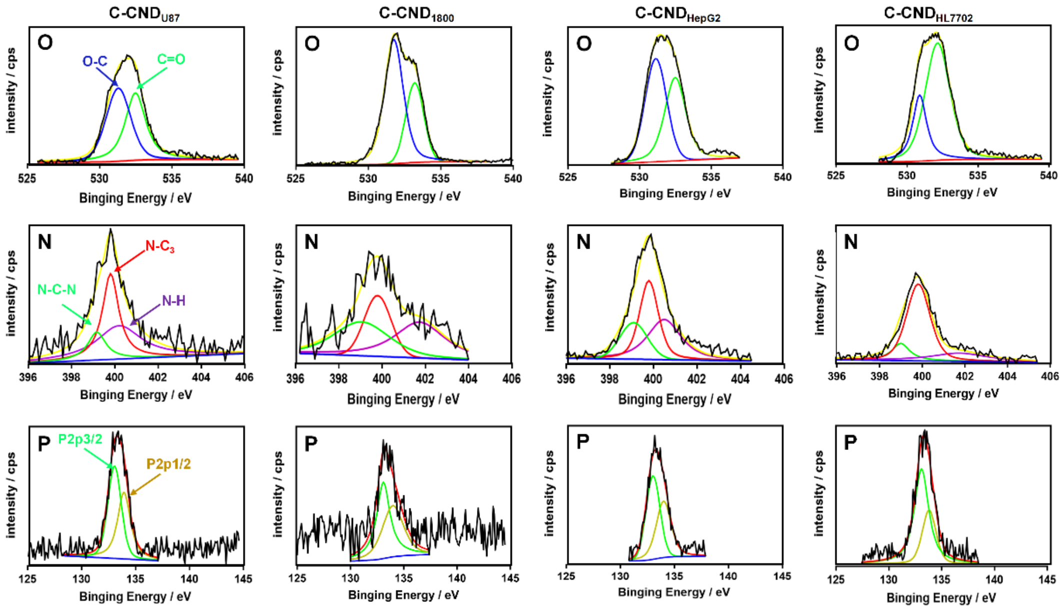

Figure S16. O1s, N1s and P1s spectra of C-CND$_{U87}$, C-CND$_{1800}$, C-CND$_{HepG2}$ and C-CND$_{HL7702}$, respectively.

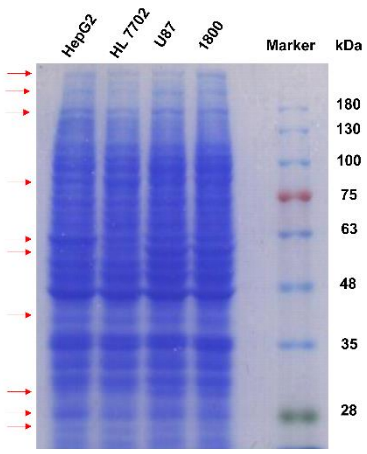

Figure S17. The SDS-PAGE analysis of proteins of U87 cell, 1800 cell, HepG2 cell and HL7702 cell. The red arrows marked the proteins with same molecular weight but varied in content between cells.

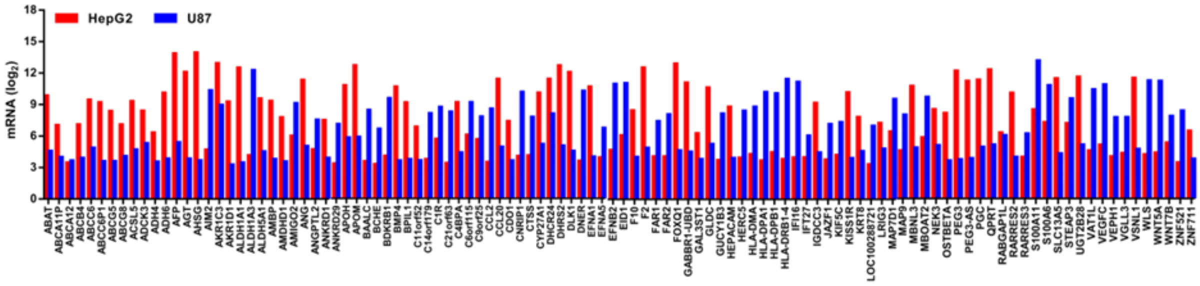

Figure S18. The differentiated mRNA expression in U87 and HepG2 cells.

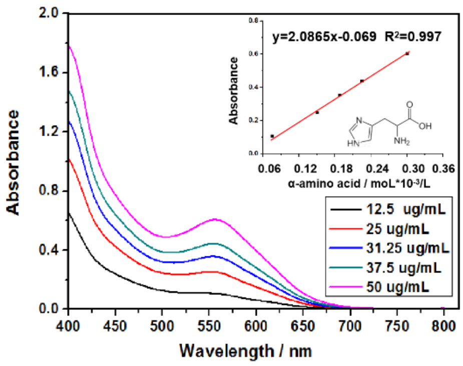

Figure S19. UV spectra of histidine after ninhydrin reaction. (Insert: Correlation of concentration and absorbance of histidine after ninhydrin reaction).

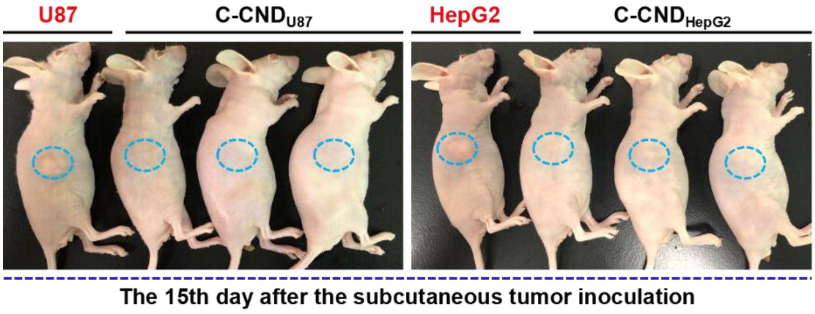

Figure S20. Tumorigenicity study of C-CND$_{U87}$ and C-CND$_{HepG2}$.

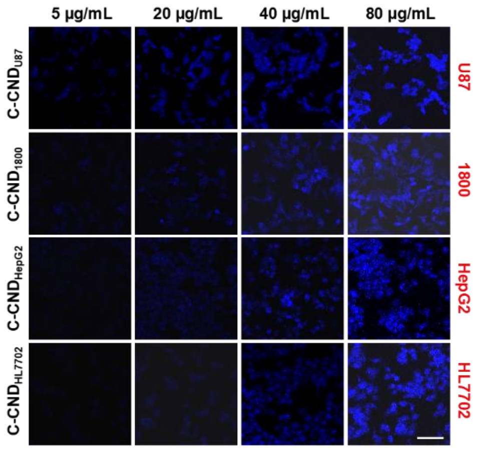

Figure S21. Confocal fluorescence images of U87, 1800, HepG2 and HL7702 cells incubated with varied concentrations of C-CND for 2 h under 405 nm excitation. Bar = 50 μm.

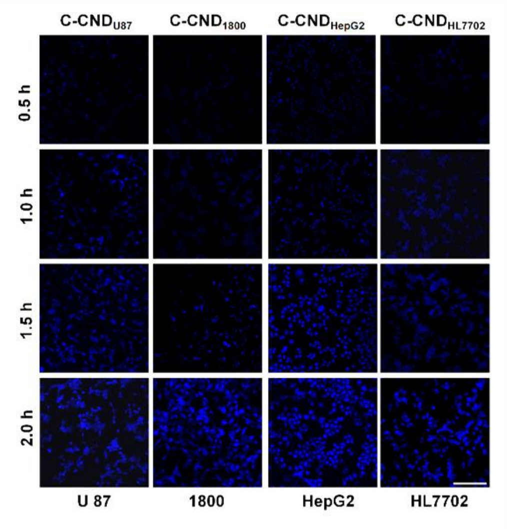

Figure S22. Confocal fluorescence images of U87, 1800, HepG2 and HL7702 cells incubated with 80 μg/mL C-CND for varied time periods under 405 nm excitation. Bar = 100 μm.

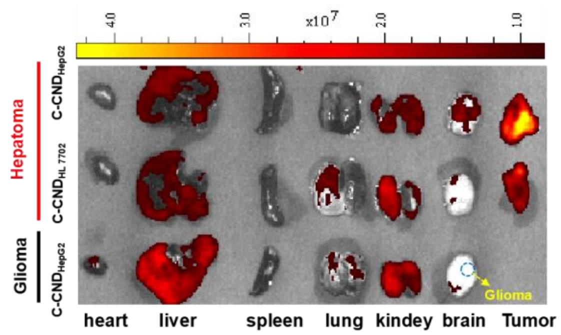

Figure S23. Ex vivo imaging of main organs of tumor-bearing mice (the subcutaneous hepatoma-bearing mice and orthotopic U87 glioma-bearing mice) collected after 3 h intravenous injection of C-CND_{HepG2} and C-CND_{HL7702}.
